# Oleuropein suppresses endometriosis progression and improves the fertility of mice with endometriosis

**DOI:** 10.1186/s12929-022-00883-2

**Published:** 2022-11-22

**Authors:** Yuri Park, Yeon Jean Cho, Nuri Sung, Mi Jin Park, Xiaoming Guan, William E. Gibbons, Bert W. O’Malley, Sang Jun Han

**Affiliations:** 1 Department of Molecular and Cellular Biology, Baylor College of Medicine, Houston, TX 77030 USA; 2 Department of Obstetrics and Gynecology, Baylor College of Medicine, Houston, TX USA; 3 Center for Reproductive Medicine, Baylor College of Medicine, Houston, TX USA; 4 Present Address: Samsung Jeil Woman’s Clinic, Busan, Republic of Korea

**Keywords:** Cytokine, Endometriosis, Estrogen receptor β, Oleuropein

## Abstract

**Background:**

Endometriosis is an estrogen-dependent inflammatory reproductive disease. Therefore, systematic estrogen depletion and anti-inflammatory drugs are the current treatment for endometriosis. However, current endometriosis treatments have low efficacy and cause adverse effects in endometriosis patients. Consequently, alternative endometriosis treatments targeting endometriosis-specific factors are in demand. In this context, ERβ was selected as a druggable target for endometriosis due to its critical role in progression. Therefore, selective targeting of ERβ without inhibiting ERα activity would be a new paradigm for endometriosis treatment to overcome the low efficacy and adverse effects of hormonal endometriosis therapy.

**Methods:**

Cell-based ERβ and ERα activity assay systems were employed to define a selective ERβ-inhibiting chemical product from a library of natural products. A surgically induced endometriosis mouse model was used to determine whether an ERβ inhibitory drug suppressed endometriosis progression. Mice with endometriosis were randomly separated and then orally treated with vehicle or 25 mg/kg oleuropein (once a day for 21 days), an ERβ inhibitory drug. The volume of endometriotic lesions or luciferase activity of endometriotic lesions was examined to define the growth of ectopic lesions in mice with endometriosis. The metabolite and levels of metabolic enzymes of the liver and kidney were determined in the serum of female mice treated with vehicle and oleuropein (25 mg/kg, once a day for 21 days) to define the toxicity of oleuropein. The in vitro decidualization assay was conducted with normal human endometrial stromal cells and endometriotic stromal cells to determine whether oleuropein overcomes decidualization in endometriosis patients. The pregnancy rate and pup numbers of C57BL/6 J female mice with endometriosis treated with vehicle or oleuropein (n = 10/group) were determined after mating with male mice. The cytokine profile in endometriotic lesions treated with vehicle and oleuropein (25 mg/kg) was determined with a Mouse Cytokine Array Kit.

**Results:**

Among natural products, oleuropein selectively inhibited ERβ but not ERα activity in vitro. Oleuropein treatment inhibited the nuclear localization of ERβ in human endometrial cells upon estradiol treatment. Oleuropein (25 mg/kg) treatment suppressed the growth of mouse (6.6-fold) and human (sixfold) ectopic lesions in mice with endometriosis compared to the vehicle by inhibiting proliferation and activating apoptosis in endometriotic lesions. Oleuropein treatment did not cause reproductive toxicity in female mice. Additionally, mice with endometriosis subjected to oleuropein treatment had a higher pregnancy rate (100%) than vehicle-treated mice (70%). Furthermore, oleuropein treatment partially recovered the decidualization impact of human endometriotic stromal cells from endometriotic lesions compared to the vehicle. Oleuropein-treated mice with endometriosis exhibited significantly lower levels of cytokines directly regulated by ERβ in ectopic lesions than vehicle-treated mice, illustrating the improvement in the hyperinflammatory state of mice with endometriosis.

**Conclusions:**

Oleuropein is a promising and novel nutraceutical product for nonhormonal therapy of endometriosis because it selectively inhibits ERβ, but not ERα, to suppress endometriosis progression and improve the fertility of mice with endometriosis.

**Supplementary Information:**

The online version contains supplementary material available at 10.1186/s12929-022-00883-2.

## Background

As an estrogen-dependent proinflammatory disease, endometriosis comprises the growth of endometrial tissues at anatomic sites outside the uterine cavity, primarily the pelvic peritoneum and ovaries [13]. Up to 15% of reproductive-aged women in the United States chronically suffer from symptoms of endometriosis, such as infertility, dysmenorrhea, and pelvic pain [13, 36].

Due to the severe chronic morbidity associated with this gynecological disorder, many past studies have attempted to identify the distinguishing molecular features of endometriotic lesions to develop more effective prognostic, diagnostic, and treatment strategies in the clinical management of this debilitating disease [13]. However, current clinical treatments are ineffective, and most yield unacceptable side effects. For example, studies have shown that prostaglandin E2 (PGE2), cyclooxygenase-2 (COX-2), and various cytokines are highly elevated in endometriotic tissue relative to their levels in normal endometrium [13, 51, 62], supporting a heightened proinflammatory response as a major component of this disease. Therefore, selective COX-2 inhibitors are often used as the first line of conventional treatment for this disorder [22, 52]. However, these inhibitors are generally ineffective and can exhibit severe off-target side effects, including potential ulcers, bleeding, perforation of the stomach and intestine, and increased risk for heart attack, stroke, and related cardiovascular conditions.

Similarly, it is well established that increased concentrations of estradiol-17β (E2) in endometriotic tissues arise from locally elevated aromatase levels and reduced activity of 17β-hydroxysteroid dehydrogenase-2 (HSD17B2) [11, 14]. Therefore, along with the anti-inflammatory treatments described above, current endometriosis treatments involve suppressing E2 levels through gonadotropin-releasing hormone agonists, oral contraceptives, synthetic progestins, and aromatase inhibitors [6]. Unfortunately, these therapies may confer adverse effects on other estrogen-targeted tissues, such as bone and brain [57]. In severe cases of endometriosis, however, total hysterectomy with bilateral salpingo-oophorectomy is the only option when inflammation is severe and estrogen suppression therapies are ineffective.

Since endometriosis is an estrogen-dependent disease, estrogen receptors (ERs) play essential roles in ectopic lesion growth. For example, ectopic lesions are not well developed in ERα- or ERβ-knockout mice [15, 30]. However, the mRNA ratio of ERβ to ERα is significantly higher in ovarian endometriomas than in normal uterine endometrium [24, 27, 35, 48, 61], suggesting that ERβ, in conjunction with high estradiol levels, plays a critical role in the development of endometriosis. Our previous study showed that ERβ interacts with the apoptosis machinery to prevent intrinsic and extrinsic apoptosis signaling in ectopic lesions, evading immune surveillance and increasing lesion survival [30]. ERβ also stimulates the inflammasome to enhance IL-1β-mediated proliferation and the adhesion of ectopic lesions. In addition, ERβ modulates TNFα/nuclear factor κB (NF-κB) signaling, epithelial-mesenchymal transition, reactive oxygen species (ROS) signaling, IL-6/Janus kinase (JAK)/signal transducer activity, activator of transcription (STAT)3 signaling, and hypoxia signaling in ectopic lesions to mediate endometriosis progression [30]. Since ERβ plays a critical role in endometriosis progression, ERβ could be considered a molecular therapeutic target for endometriosis treatment.

Natural products have been widely and globally used in various preventive and therapeutic health care formats. Unlike synthetic compounds, natural products are generated by enzymatic interactions. The biological activity of natural products involves protein‒protein binding, making them more effective drug candidates. In addition, natural products are a product of evolutionary pressure that results in their novelty. Therefore, natural products are more prone to bioactivity than synthetic compounds [41]. For example, 48.6% of cancer drugs have natural origins or are derived from natural products [16]. Therefore, natural products could also be employed to treat endometriosis as nonhormonal therapies.

Here, natural product screening revealed that oleuropein selectively inhibits ERβ activity and suppresses the progression of endometriosis in mice. Olive leaf extract has protective effects against the reproductive toxicity of lead acetate in rats [2]. In addition, olive leaf extracts have various beneficial effects on human health, such as antimicrobial, antiviral, antioxidant, anti-inflammatory, antiaging-associated neurodegeneration, and anticancer effects [9, 10, 17, 65]. Oleuropein is a major component of olive leaves [up 19% (w/w)] [42]. Oleuropein is metabolized in vivo into elenolic acid and hydroxytyrosol by β-glucosidase and esterase activity in humans and mice [53]. Hydroxytyrosol is also one of the major phenolic components in olive leaf extracts and has antiproliferative, antioxidant, and anti-inflammatory effects on various human cancers [18, 60, 63]. Therefore, we propose that oleuropein represents a new nutraceutical product for the naturopathy of endometriosis.

## Material and methods

### Mice

C57BL/6J, luciferase-expressing FVB [Tg(CAG-luc, GFP)L2G85Chco], FVB, and SCID female mice were purchased from Jackson Laboratory and maintained in the designated animal care facility at Baylor College of Medicine according to the Institutional Animal Care and Use Committee (IACUC) guidelines for the care and use of laboratory animals. An IACUC-approved protocol was followed for all animal experiments in this study.

### Material

Oleuropein was purchased from Santa Cruz Biotechnology (catalog number: CAS 32619-42-4), and 4-[2-phenyl-5,7-*bis*(trifluoromethyl)pyrazolo[1,5-*a*]pyrimidin-3-yl]phenol (PHTPP) was purchased from Tocris Bioscience (catalog number: 2662).

### Transfection and luciferase reporter gene assay

According to the manufacturer's instructions, the transfection of cells with plasmids was performed using Lipofectamine 2000 (ThermoFisher, catalog number: 116680300). *HeLa* cells were transfected with the indicated expression plasmids. The natural product library was purchased from Selleckchem (catalog number: L1440). After 24 h, phenol red-free Dulbecco's minimum essential medium (DMEM) containing 10% charcoal-stripped fetal bovine serum (FBS) was added to the cells. Estradiol (10^−8^ M) and estradiol (10^−8^ M) plus natural product (10^−8^ M) were added to the cells 24 h after the medium was changed and incubated for another 24 h. The cells were harvested, and the luciferase activity was determined and normalized against the total input protein levels.

### Generation of firefly luciferase-labeled immortalized human endometrial epithelial cells (IHEECs) and immortalized human endometrial stromal cells (IHESCs)

For the noninvasive assay of ectopic lesion growth in mice with endometriosis, the firefly luciferase gene was cloned into the pSMPUW-Hygro construct (Cell Biolabs, catalog number: VPK-214). Lentivirus carrying luciferase gene were generated 293 T cells by transfection with pSMPUW-Hygro containing luciferase gene and the Lenti-X high-titer lentiviral packaging system (ClonTech, catalog number: 631278). The recombinant lentivirus titer was measured using Lenti-X™ GoStix™ Plus (ClonTech, catalog number: 631280). IHEECs and IHESCs were transduced with lentiviral vectors carrying the luciferase expression cassette with TransDux MAX™ (System Bioscience, catalog number: LV860A-1). Luciferase-labeled IHEECs and IHESCs were then selected in the presence of 300 µg/ml hygromycin. The luciferase gene expression in these recombinant cells was validated using a luciferase activity assay kit (Promega, catalog number: E1980). All these recombinant cells were maintained with DMEM/F12 supplemented with 10% FBS and penicillin/streptomycin under drug selection.

### Surgically induced endometriosis

Endometriosis in mice was surgically induced under aseptic conditions and anesthesia [30]. A) *Autotransplantation*: C57BL/6 female mice (8 weeks old) were subjected to ovariectomy. After one week, the ovariectomized mouse was implanted with a sterile, 60-day release pellet containing 0.36 mg of 17-β estradiol (Innovative Research of America). One day later, one uterine horn of each mouse was isolated under anesthesia. In a Petri dish containing warmed DMEM/F-12 supplemented with 100 U/ml penicillin and 100 µg/ml streptomycin, the uterine horns were longitudinally cut with scissors. Next, using a 2-mm dermal biopsy punch (Miltex), one endometrial fragment was isolated and subsequently sutured to the mesenteric membrane attached to the intestine in the same mouse through a midline incision (7–0 braided polypropylene suture; Ethicon). The abdominal incision was closed continuously with a 5–0 braided polypropylene suture (Ethicon).

(B) *Heterotransplantation with luciferase-labeled endometrial tissue*: FVB female mice (8 weeks old) were ovariectomized. After one week, the ovariectomized mouse was implanted with a sterile, 60-day release pellet containing 0.36 mg of 17-β estradiol. The uterus was isolated from luciferase-expressing FVB [Tg(CAG-luc, GFP)L2G85Chco] female mice (8 weeks old) at the proestrus stage because endometriosis-like lesions were found to be 1.9 times more likely to establish in proestrus than in estrus [20]. One uterine horn was fragmented (~ 1mm^3^) with scissors and resuspended in 0.5 ml of sterilized PBS. The 0.5 ml of the luciferase-labeled endometrial fragment was intraperitoneally injected into ovariectomized FVB female mice implanted with an estrogen pellet. After endometriosis induction, bioluminescence images of the ectopic lesion were analyzed using an In Vivo Image Analysis System (IVIS, Xenogen).

(C) *Heterotransplantation with cultured human endometrial cells*: Luciferase-labeled IHESCs and luciferase-labeled IHEECs were cultured in DMEM/F12 containing 10% FBS and penicillin (100 U/mL), streptomycin (100 mg/mL), amphotericin-B (2.5 mg/mL), and 300 µg/ml hygromycin in humidified 5% CO2 and 95% air at 37 °C. The medium was changed every other day. On the day of transplantation, the cells were trypsinized with 0.1% trypsin-ethylenediaminetetraacetic acid. Luciferase-labeled IHESCs (2 × 10^6^ cells) were mixed with luciferase-labeled IHEECs (2 × 10^6^ cells) with 100 µl of DMEM/F12 and then mixed with 100 µl of Matrigel (BD Biosciences) at a 1:1 ratio. The cell suspension mixture with Matrigel (200 µl) was administered intraperitoneally on the midventral line just caudal to the umbilicus of ovariectomized severe combined immunodeficiency (SCID) female mice (8 weeks old) implanted with a sterile 60-day release pellet containing 0.36 mg of 17-β estradiol. After the induction of endometriosis, bioluminescence images were analyzed twice a week using an In Vivo Image System (IVIS, Xenogen).

### Quantifying bioluminescence data

Mice were anesthetized with a 1.5% isoflurane/air mixture using an Inhalation Anesthesia System (VetEquip). D-Luciferin (ThermoFisher, catalog number: L2916) was intraperitoneally injected at 40 mg/kg mouse body weight. Ten minutes after the D-luciferin injection, the mice were imaged using an IVIS Imaging System (Xenogen) with continuous 1 to 2% isoflurane exposure. Imaging variables were maintained for comparative analysis. Grayscale-reflected and pseudocolorized images reflecting bioluminescence were superimposed and analyzed using Living Image software (Version 4.4, Xenogen). A region of interest (ROI) was manually selected over the relevant signal intensity regions. The area of the ROI was kept constant across experiments, and the intensity was recorded as total photon counts per second per cm^2^ within the ROI.

### Weight measurement

C57BL/6 J female mice (8 weeks old) were randomly divided into two groups (n = 5/group) and intraperitoneally treated with oleuropein (25 mg/kg) or vehicle (corn oil) for 21 days. Body weight was measured 3 times per week for 21 days.

### Liver panel assay

C57BL/6J female mice (8 weeks old) were intraperitoneally treated with oleuropein (25 mg/kg, n = 3) and vehicle (corn oil, n = 3) once a day for 21 days. Afterward, we collected whole blood and allowed the blood to clot at room temperature for 20 min. Next, we removed the clot by centrifuging at 1000–2000×*g* for 10 min in a refrigerated centrifuge and then collected the supernatant as serum. In the liver panel assay, the levels of total protein (TP), alanine aminotransferase (ATL), aspartate aminotransferase (AST), alkaline phosphatase (ALP), total bilirubin (TBIL), direct bilirubin (DBIL), and indirect bilirubin (IBIL) and the albumin-globulin ratio (A/G) in serum were determined by the Clinical Pathology Core of the Center for Comparative Medicine at Baylor College of Medicine.

### Oleuropein treatment in mice

Oleuropein was dissolved in corn oil (100 mg/ml). Female mice with endometriosis generated by heterotransplantation with luciferase-labeled endometrial tissue (21 days after induction) were randomly separated into three groups (n = 4/group) and then orally treated with corn oil, 25 mg/kg, and 200 mg/kg oleuropein once a day for 31 days.

### Reproductive toxicity analysis for oleuropein

C57BL/6J female mice (8 weeks of age) were orally treated daily with vehicle and oleuropein (25 mg/kg) for 21 days (n = 5/group). Afterward, each female mouse was paired with a wild-type male of proven fertility (1:1). Fertility was assessed by monitoring the litter size and pregnancy rate.

### Effects of oleuropein on the pregnancy rate of mice with endometriosis

Endometriosis was induced in mice using the heterotransplantation method. Briefly, the uterus was isolated from donor C57BL/6J female mice (8 weeks old) at the proestrus stage and then fragmented (~ 1mm^3^) with scissors. Endometrial fragments from one uterine horn were resuspended in 0.5 ml of sterilized PBS and injected into one wild-type recipient ovary-intact C57BL/6J female mouse (8 weeks old). At 3 days after the injection of endometrial fragments, female mice with endometriosis were randomly separated into two groups (n = 10/group) and then orally treated with corn oil or 25 mg/kg oleuropein once a day for 21 days. At the same time, each female mouse was paired with a wild-type male of proven fertility (1:1). Fertility was assessed by monitoring the litter size and pregnancy rate. Wild-type C57BL/6J female mice (8 weeks old) without endometriosis (n = 5) were used as the control group.

### Immunohistochemistry

Immunostaining was performed with 10% neutral-buffered, formalin-fixed and paraffin-embedded sections of mouse ectopic lesions. Antibodies against Ki-67 (Abcam, catalog number: ab16667) and cleaved caspase 3 (Cell Signaling, catalog number: 9661) were used. The specific antigens were visualized with a DAB substrate kit (Vector, catalog number: SK-4100). The immunostaining intensity was quantified using QuPath software [7].

### Immunofluorescence analysis of subcelluar localization of ERβ in ERβ-overexpressing immortalized human endometrial epithelial cells (IHEECs: ERB) upon oleuropein treatment

IHEECs: ERB cells were cultured in DMEM/F12 containing 10% fetal bovine serum (FBS). When the cell confluence was 90%, the IHEEC:ERB cells were washed with phosphate-buffered saline (PBS), and phenol-red free DMEM/F12 containing 10% charcoal-stripped FBS was added. Two days later, the IHEECs:ERB were cultured with phenol-red free DMEM/F12 containing 10% charcoal-stripped FBS in the presence of vehicle, 10 nM estradiol, 10 nM oleuropein, or 10 nM estradiol plus 10 nM oleuropein for 24 h. Afterward, the IHEECs:ERB cells were fixed with 4% paraformaldehyde, permeabilized with 0.3% Triton X-100, blocked, and incubated with primary antibodies against ERβ (Abcam, catalog number: ab16813, 1:500) at 4 °C overnight. Subsequently, the IHEECs:ERB cells were stained with appropriate goat anti-mouse IgG Alexa 488 (Invitrogen, catalog number A11001, 1:500). Nuclei was stained with Hoechst 33442 (Sigma, catalog number: B2261). Images were taken with Zeiss Axiocam 202 mono using Zeiss 3.1 blue edition software.

### 3-(4,5-Dimethylthiazol-2-yl)-5-(3-carboxymethoxyphenyl)-2-(4-sulfophenyl)-2H-tetrazolium) (MTS) cell growth assay

Human endometrial cells were inoculated into 96-well plates (1 × 10^4^ cells/well). The next day, each cell line was treated with serially diluted oleuropein or PHTPP. After 3 days, 10 μL of MTS reagent (Promega catalog number: G1111) was added to each well, and the MTS-treated plates were incubated for 2 h. Then, the optical density of the color in each well was measured at 450 nm using a microtiter plate reader.

### Determination of the proliferation of ERβ-knockdown endometriotic HESCs upon oleuropein treatment

Endometriotic HESCs from endometriosis patients [49] were plated into 96-well plates. When the cells were 70% confluent, they were treated in triplicate with Lipofectamine 2000 (Invitrogen Corporation) and 1 nM (final concentration) of mixed ERβ siRNA (SR301462A rGrGrCrArArCrUrArCrUrUrCrArArGrGrUrUrUrCrGrArGAG, SR301462B rCrUrArCrArArUrCrArGrUrGrUrArCrArArUrCrGrArUAA, SR301462C rGrCrArArUrGrUrCrArCrUrArArCrUrUrGrGrArArGrGrGrUGG, Origene). As the control, HESCs were treated with 1 nM scramble (sc) siRNA (Invitrogen, catalog number: AM4611). After 72 h, the HESCs were treated with different oleuropein doses for 3 days. Then, the MTS assay was performed to measure the proliferation activity of HESCs treated with ERβ siRNA versus sc siRNA.

### Human phospho kinase array

Endometriotic HESCs were treated with vehicle or 10 or 100 nM oleuropein for 24 h. Then, the levels of phospho kinases in these cells were determined with the Human Phosphorylation Pathway Profiling Array C55 kit (RayBiothech, catalog number: AAH-PPP-1-8).

### In vitro decidualization

We previously generated primary human endometrial stromal cell lines and human endometriotic stromal cell lines from ovarian endometrioma [49]. These human endometrial stromal cells were cultured in six-well plates (1 × 10^5^ cells per well in triplicate) with DMEM/F-12 media containing 10% FBS, 100 units/ml penicillin, and 0.1 mg/ml streptomycin. At 90% confluence, the human endometrial cells were cultured with 1 × Opti-MEM I reduced-serum medium containing 2% FBS, 100 units/ml penicillin, and 0.1 mg/ml streptomycin. After 24 h, the human endometrial cells were treated with 1 × Opti-MEM I reduced-serum media with 2% FBS plus decidualization hormone cocktail [EPC; estradiol (100 nM), medroxyprogesterone acetate (MPA: 10 µM, Sigma–Aldrich) and cAMP (50 µM, Sigma–Aldrich)]. The day that the decidualization medium was added to the human endometrial cells was designated Day 0. For these studies, the decidualization medium was renewed every other day. Cells were harvested on the 3rd day after adding the decidualization hormone cocktail. Total RNA was isolated to assess the transcript levels of the decidualization markers prolactin (PRL) and insulin-like growth factor binding protein-1 (IGFBP-1) [12].

### Cytokine/chemokine analysis of mice with ectopic lesions

Endometriosis was induced in C57BL/6J female mice (8 weeks old) with the autotransplantation method. On the 21st day after endometriosis induction, the mice were randomly separated into two groups (n = 4/group). The mice in Group 1 were orally treated with corn oil, and the mice in Group 2 were orally treated with 25 mg/kg oleuropein once a day for 31 days. Afterward, ectopic lesions were isolated, and the cytokine and chemokine levels in the ectopic lesions were determined using a Proteomic Profiler Mouse Cytokine Array Kit (R&D System, ARY0066). The cytokine levels were quantified with ImageJ software [56].

### Western blot analysis

Primary antibodies against the following proteins were used: FLAG (Sigma, catalog number: F7425), ERβ (Abcam, catalog number: ab288), and tubulin (Santa Cruz Biotechnology, catalog number: sc-8035). In addition, membranes containing proteins were incubated with secondary HRP-tagged antibodies (Abcam, catalog number:ab6721), and the signals were visualized using SuperSignal™ West Pico Plus Chemiluminescent substrate (ThermoFisher, catalog number:34580).

### FLAG-ERβ chromatin immune-precipitation sequencing (ChIP-Seq)

Tissue was submersed in PBS + 1% formaldehyde, cut into small pieces, and incubated at room temperature for 15 min. The addition of 0.125 M glycine stopped fixation. The tissue pieces were then treated with a Tissue Tearer, spun down and washed 2 × in PBS. Chromatin was isolated by the addition of lysis buffer, followed by disruption with a Dounce homogenizer. The lysates were sonicated, and the DNA sheared to an average length of 300–500 bp. For each ChIP reaction, 50 µg of precleared chromatin was mixed with Flag M2 agarose and incubated for three hours. The immune complexes were washed, eluted from the beads with SDS buffer, and subjected to RNase and proteinase K treatment. Crosslinks were reversed by incubation overnight at 65 °C, and ChIP DNA was purified by phenol‒chloroform extraction and ethanol precipitation. Illumina sequencing libraries were prepared from the ChIP and input DNA by the standard consecutive enzymatic steps of end polishing, dA addition, and adaptor ligation. After a final PCR amplification step, the resulting DNA libraries were quantified and sequenced on a NextSeq 500. Standard Illumina software base-calling and quality-control filtering were applied. Sequences (75 nt reads, single-end) were aligned to the mouse genome (mm10) using the BWA algorithm (v0.7.12, default parameters). Alignments were extended in silico at their 3’-end to a length of 200 bp, which was the average genomic fragment length in the size-selected library, and assigned to 32-nt bins along the genome. The resulting histograms (genomic “signal maps”) were stored as bigWIG files. Peak locations were determined using the MACS algorithm (v2.1.0) with a cutoff of p = 1E−7. Signal maps and peak locations were used as input data to the Active Motifs proprietary analysis program, which creates Excel tables containing detailed information on sample comparison, peak metrics, peak locations, and gene annotations. The FLAG-ERβ ChIP sequencing was performed by Active Motif (Active Motif, CA). The GEO accession number for the ER β-cistrome is GSE114047.

### Statistical analysis

An independent two-tailed Student's t test was used to assess the statistical significance with GraphPad Prism version 8.0. P < 0.05 was considered statistically significant.

## Results

### Oleuropein selectively inhibited ERβ activity but not ERα activity

**Fig. 1:**
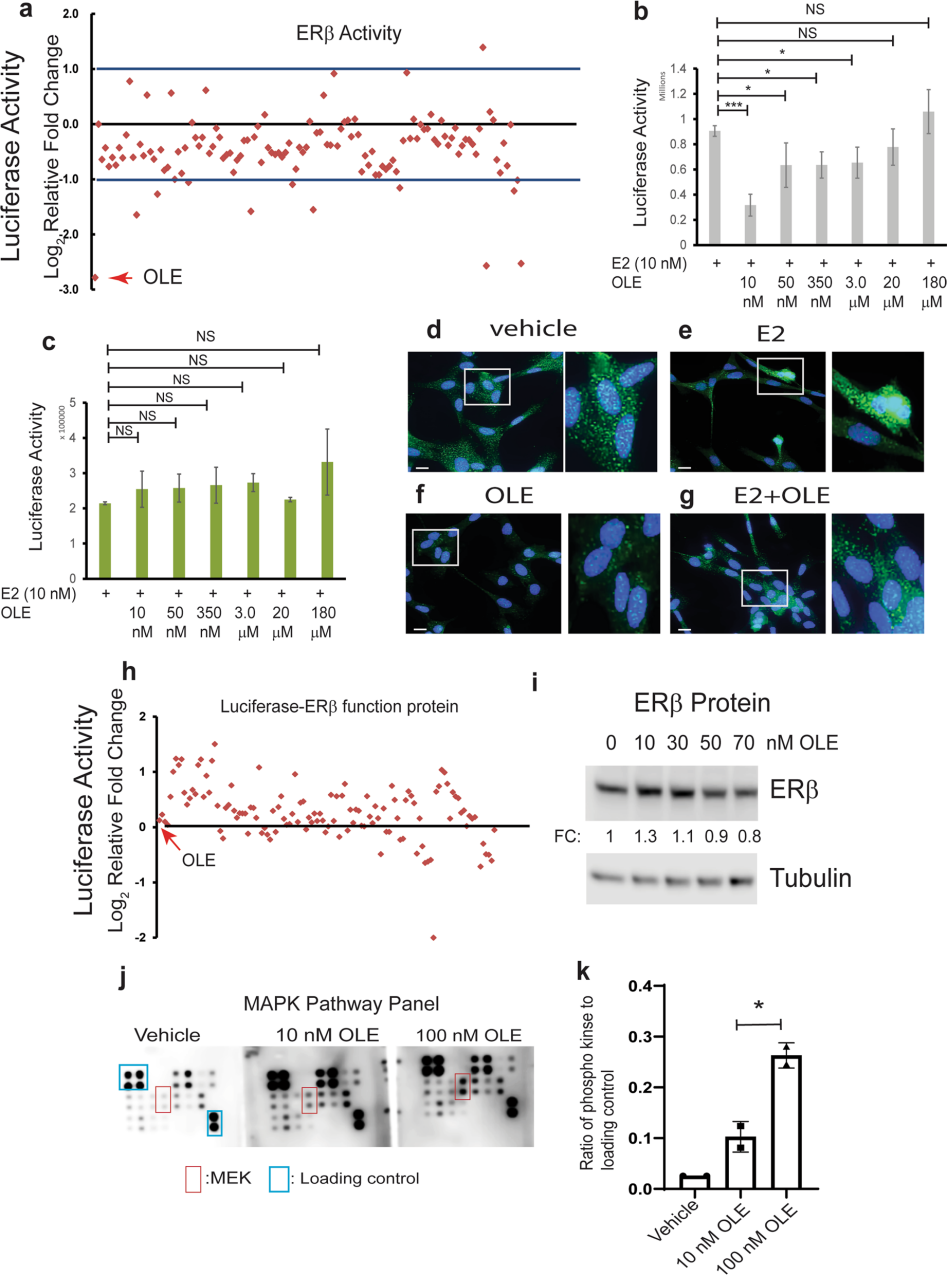
Inhibition of ERβ activity by oleuropein **a** Natural product screening for ERβ-selective inhibitors. *HeLa* cells were transiently transfected with ERβ expression vector and ERE-luciferase reporter. The ratio of luciferase activity upon E2 (10 nM) administration plus natural product (10 nM) or E2 (10 nM) alone was determined. Oleuropein (10 nM) significantly inhibited ERβ activity compared with the vehicle (arrowhead). **b**, **c** Dose-dependent effect of oleuropein on ERβ and ERα activity. *HeLa* cells were transiently transfected with ERβ (**b**) or ERα (**c**) expression vector plus ERE-luciferase reporter. The luciferase activity was determined upon treatment with E2 (10 nM) plus various concentrations of oleuropein. **d-g** Immunofluorescence staining of ERβ in IHEECS:ERB cells upon treatment with the vehicle (**d**), 10 nM E2 (**e**), 10 nM oleuropein (**f**), and 10 nM E2 plus 10 nM oleuropein (**g**) for 24 h. **h** Screening of natural products for ERβ protein degradation. *HeLa* cells were transiently transfected with an expression plasmid for the luciferase-ERβ fusion protein, and the ratio of luciferase activity after treatment with natural products (10 nM) or the vehicle was determined at 24 h posttreatment. The arrowhead indicates oleuropein. **i** Effect of oleuropein on ERβ protein stability. Primary human endometriotic stromal cells were treated with different doses of oleuropein, and ERβ protein levels were determined by Western blot analysis at 24 h after treatment. The tubulin level determined the protein loading amount el. **j** Expression profile of phospho kinases involved in MAPK pathways in ectopic HESCs treated with vehicle or 10 and 100 nM OLE for 24 h. **k** Quantification of the ratio of phospho-MEK to the loading control in Panel j. OLE, oleuropein. *, p < 0.05; **, P < 0.01; ***, p < 0.001; NS, nonspecific. Scale bar is 200 μm

Since ERβ has an essential role in endometriosis progression, ERβ-specific inhibition might effectively suppress endometriosis progression without the adverse effects of ERα inhibition in women. To screen ERβ-specific drugs, we employed a natural product library because most natural products are safer and cause fewer side effects than synthetic drugs [1]. To determine ERβ activity, *HeLa* cells were transfected with an ERβ expression plasmid and ERE-luciferase reporter plasmid. The ERβ activity was determined by luciferase activity upon estradiol (10 nM) treatment with 10 nM of natural products versus estradiol (10 nM) alone. The comparative analysis of luciferase activity between estrogen and estrogen plus natural products revealed that oleuropein-treated cells had significantly less ERβ activity than vehicle-treated cells (Fig. 1a, b). However, the cells treated with a higher concentration of oleuropein did not have substantially lower ERβ activity than the vehicle-treated cells (Fig. 1b). In addition to ERβ, we determined whether oleuropein inhibits ERα activity with a transfection assay using an ERα expression plasmid and assessment of ERE-luciferase activity in *HeLa* cells. In contrast with ERβ, oleuropein (10 nM)-treated cells did not exhibit significantly lower ERα activity than the vehicle-treated cells (Fig. 1c). In addition, a high concentration of oleuropein (180 µM) did not impact ERα activity (Fig. 1c). Therefore, oleuropein selectively inhibited ERβ but not ERα activity.

How does oleuropein inhibit ERβ activity? To address this question, the subcellular location of ERβ in immortalized human endometrial epithelial cells overexpressing ERβ (IHEECs:ERΒ) [30] was determined upon treatment with vehicle, estradiol (10 nM), oleuropein (10 nM) or estradiol plus oleuropein for 24 h. In the absence of estradiol, the immunofluorescence results reveal that most ERβ was located in the cytoplasm (Fig. 1d). Estradiol treatment induced the nuclear localization of ERβ (Fig. 1e), but oleuropein did not enhance the nuclear localization of ERβ in human endometrial epithelial cells (Fig. 1f). Moreover, oleuropein effectively suppressed the ERβ nuclear localization induced by estradiol in human endometrial epithelial cells (Fig. 1g).

To screen the natural products that reduce ERβ protein levels, we generated a luciferase-ERβ fusion protein expression vector based on a previous study because the luciferase activity of the luciferase-ERβ fusion protein represented the levels of ERβ protein in vivo [45]. The ERβ protein stability assay revealed that oleuropein treatment did not affect the luciferase activity (Fig. 1h). Therefore, oleuropein did not alter ERβ protein levels. To validate this observation, human endometrial stromal cells from endometriosis patients, named human endometriotic stromal cells, were treated with different doses of oleuropein because these cells have a higher level of ERβ than endometrial stromal cells from women without endometriosis [30]. Oleuropein-treated and vehicle-treated human endometriotic stromal cells did not exhibit different ERβ protein levels (Fig. 1i). Collectively, the results indicate that oleuropein inhibited ERβ activity without ERβ protein degradation.

To determine why high doses of oleuropein did not inhibit ERβ activity, we determined the phospho-kinase levels in endometriotic human endometrial stromal cells upon treatment with different doses of oleuropein because oleuropein activates intracellular kinase activity, and activated kinase signaling also impacts ERβ activity [32, 66]. The 10 nM oleuropein activated several kinases involved in MAPK pathways compared to the vehicle in human endometrial stromal cells (Fig. 1j). Compared to 10 nM oleuropein, 100 nM oleuropein significantly activated mitogen-activated protein kinase kinase (MEK) in human endometrial stromal cells (Fig. 1k). Compared to the MAPK pathway, oleuropein did not activate the JAK/STAT, NF-κB and TGFβ signaling pathways in human endometrial stromal cells upon treatment with 10 and 100 nM oleuropein compared to the vehicle (Additional file 1). In contrast with MAPK kinase, therefore, different doses of oleuropein did not activate kinases involved in AKT, JAK/STST, NF-κB, and TGFβ signaling pathways. MEK is involved in estrogen receptor activation [5]. Therefore, activation of MEK signaling in human endometrial stromal cells by high doses of oleuropein partly explains why a high dose of oleuropein (100 nM) may not effectively suppress ERβ activity compared to a low dose of oleuropein (10 nM).

### Oleuropein selectively suppressed the growth of human endometrial cells exhibiting high levels of ERβ

**Fig. 2:**
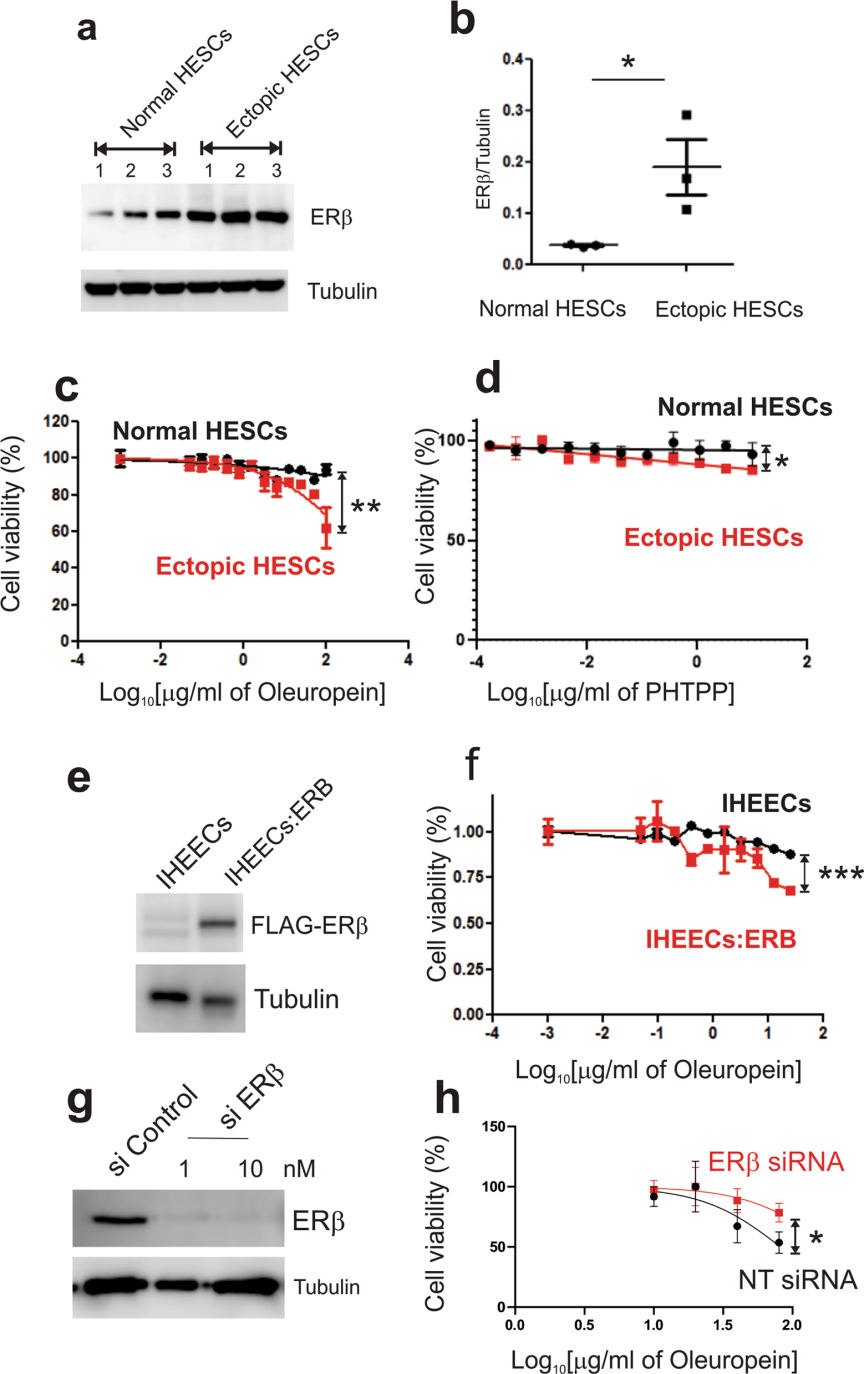
Selective inhibition of human endometrial cell growth with a high level of ERβ. **a** Elevated levels of ERβ in human endometriotic stromal cells (Ectopic HESCs) from ectopic lesions of endometriosis patients compared to normal human endometrial stromal cells (Normal HESCs) determined by Western blot. Tubulin levels were determined to normalize the protein loading amount. **b** Quantification of the ERβ levels in Panel **a**. **c** The growth inhibition of ectopic HESCs, but not normal HESCs, by oleuropein, showing the effects of the dose of oleuropein on the viability of ectopic HESCs and normal HESCs. **d** The growth inhibition of ectopic HESCs and normal HESCs by PHTPP, showing the effects of PHTPP on the viability of ectopic HESCs and normal HESCs. **e** ERβ overexpression in immortalized human endometrial epithelial cells (IHEECs). FLAG tagged ERβ expression levels were determined in ERβ-overexpressing IHEECS (IHEECs: ERB) and parental IHEECs by Western blot analysis. **f** The growth inhibition of IHEECs:ERB and IHEECs by oleuropein, showing the cell viability of IHEECs:ERB and IHEECs with various doses of oleuropein. **g** Reduction in ERβ protein levels in ectopic HECS treated with 1 and 10 nM ERβ siRNA compared to Non-Target (NT) siRNA control determined by Western blot analysis. **h** ERβ siRNA suppressed the oleuropein-mediated growth inhibition of ectopic HESCs compared to NT siRNA. *, p < 0.05; **, P < 0.01; ***, p < 0.001

We generated primary human endometrial stromal cell lines from women without endometriosis (Normal HESCs) and primary human endometriotic stromal cell lines from ovarian endometrioma (Ectopic HESCs) [49]. Western blotting analysis revealed that human endometriotic stromal cells had a higher level of ERβ than human normal endometrial stromal cells (Fig. 2a, b).

Next, we examined whether oleuropein selectively suppresses the growth of human endometriotic stromal cells over human normal endometrial cells. The IC_50_ value of oleuropein for MCF-7 cells was 200 to 400 µg/mL [26]. Based on this result, human endometrial stromal cells were treated with different concentrations of oleuropein. Oleuropein (200 µg/ml) effectively suppressed the viability of human endometriotic stromal cells to 32.7% of the amount observed in the vehicle-treated group (Fig. 2c). However, oleuropein (200 µg/ml) decreased the viability of normal human endometrial stromal cells to only 5.4% of the amount observed in the vehicle-treated group (Fig. 2c). Therefore, oleuropein selectively and significantly suppresses the growth of human endometriotic stromal cells over normal human endometrial stromal cells due to a high level of ERβ. Our previous study revealed that PHTPP, a selective ERβ antagonist, suppressed endometriosis progression in mice [30]. Treatment with 20 µM (8.6 µg/ml) PHTPP reduced 50% of the variability in breast cancer cells [34]. Based on this IC50 value, human endometrial stromal cells were treated with different doses of PHTPP. However, PHTPP (10 µg/ml) reduced the viability of human endometriotic stromal cells and normal human endometrial stromal cells by 14.8% and 7.4%, the amounts observed in oleuropein-treated cells, respectively (Fig. 2d). Therefore, oleuropein has a better inhibition efficiency of human endometriotic stromal cell growth than PHTPP.

To define the effect of oleuropein on the proliferation of human endometrial epithelial cells, we employed immortalized human endometrial epithelial cells overexpressing ERβ (IHEECs:ERΒ) and their parental IHEECs as the control [30]. Exogenous FLAG-tagged ERβ was expressed in IHEECs:ERΒ, unlike IHEECs (Fig. 2e). The oleuropein treatment inhibited 12.0% of the viability of parental IHEECs, but 32.6% of the viability of IHEECs:ERΒ was inhibited by oleuropein (Fig. 2f). Therefore, the elevation of ERβ in human endometrial cells increased the sensitivity of the growth inhibitory effect of oleuropein.

Next, we determined whether ERβ has a key role in oleuropein-mediated growth suppression of ectopic HESCs. ERβ protein levels were downregulated by ERβ siRNA in ectopic HESCs. The 1 nM ERβ siRNA effectively downregulated ERβ protein levels in HECS compared to Non-Target (NT) siRNA (si Control) (Fig. 2g). Then, we determined the growth suppressive effect of oleuropein on ectopic HESCs treated with 1 nM ERβ siRNA. Compared to NT siRNA, ERβ siRNA significantly prevented the oleuropein-mediated growth suppression of HESCs (Fig. 2h). Therefore, ERβ is the critical factor required for the growth suppression of ectopic HESCs by oleuropein.

### Oleuropein effectively suppressed the growth of mouse and human ectopic lesions in mice

**Fig. 3:**
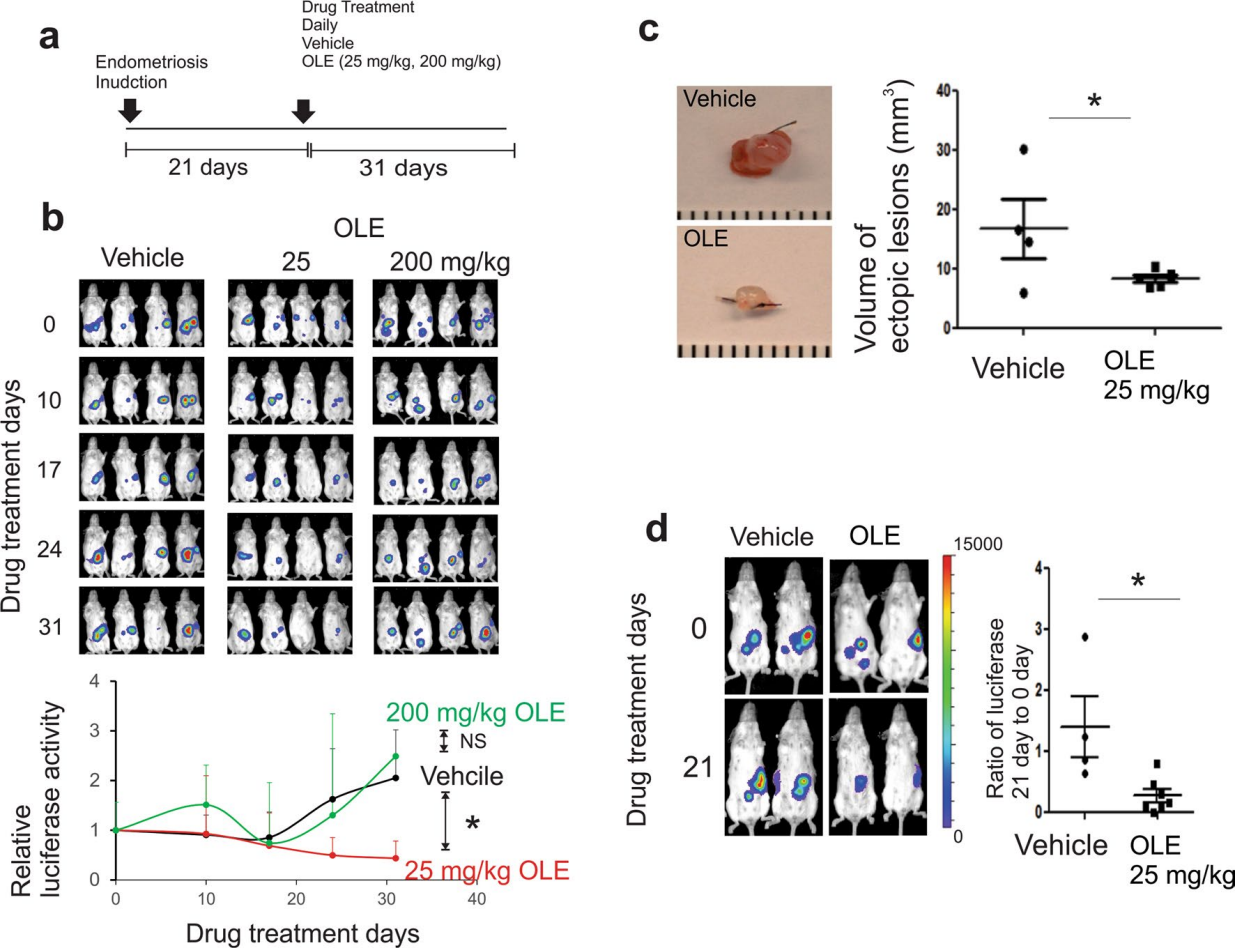
Suppression of the growth of mouse and human ectopic lesions in mice with endometriosis by oleuropein. **a** Oleuropein treatment plan. After ectopic lesions were established in different mouse models (21 days), the mice were randomly separated and then orally treated with vehicle or oleuropein (25 mg/kg or 200 mg/kg, once a day, 31 days). **b** Inhibition of mouse ectopic lesion progression in mice with endometriosis using heterotransplantation with luciferase-labeled endometrial tissues. Oleuropein treatment (25 mg/kg) significantly reduced the luciferase activity of ectopic lesions in mice with endometriosis. However, 200 mg/kg oleuropein did not suppress luciferase activity compared to the vehicle. **c** Reduction in the volume of ectopic lesions by oleuropein. Endometriosis was induced in mice with the autotransplantation method. Ectopic lesions were isolated from mice with endometriosis after 31 days of oleuropein or vehicle treatment. The volume of ectopic lesions was determined using the modified ellipsoid Formula 1/2(Length × Width^2^) [23]. **d** Inhibition of the progression of human ectopic lesions by oleuropein. Human ectopic lesions were generated in SCID female mice by heterotransplantation with the cultured human endometrial cell method. After establishing the human ectopic lesion (21 days after endometriosis induction), SCID mice with endometriosis were treated with oleuropein (25 mg/kg, once a day, 21 days) or vehicle. Oleuropein treatment significantly reduced the luciferase activity of human ectopic lesions. OLE, oleuropein. *, P < 0.05

Endometriosis was induced by heterotransplantation of endometrial fragments into the peritoneal cavity in ovariectomized FVB female mice bearing an estrogen pellet. The tumor volumes of mice treated with extracts of olive leaves (300 and 1000 mg/kg body weight) and oleuropein (25 mg/kg body weight) were notably reduced in weeks 25 to 30 [38]. Based on this observation, mice with endometriosis were orally treated with oleuropein (25 and 200 mg/kg) and vehicle (control) once a day for 31 days after the establishment of ectopic lesions in mice (21 days after endometriosis induction) (Fig. 3a). The 25 mg/kg oleuropein treatment led to significantly lower luciferase activity (6.6-fold, p = 0.02) of ectopic lesions than vehicle treatment (Fig. 3b). However, 200 mg/kg oleuropein did not lead to significantly lower luciferase activity of ectopic lesions than the vehicle (Fig. 3b). To further validate the effects of oleuropein on endometriosis, endometriosis was induced in C57BL/6J mice by autotransplanting the endometrial fragment into the mesentery membrane of the intestine by suturing. Then, oleuropein was administered as described in Panel a. Ectopic lesions were isolated from the mice with endometriosis after the final drug treatment. Consistent with the luciferase activity, 25 mg/kg oleuropein treatment (8.5 mm^3^) led to significantly smaller ectopic lesions than vehicle treatment (19.0 mm^3^) (Fig. 3c).

In addition to mouse ectopic lesions, we determined the effect of oleuropein on the suppression of the progression of human ectopic lesions. To induce human ectopic lesions in mice, a mixture of luciferase-labeled immortalized human endometrial stromal cells and luciferase-labeled immortalized human endometrial epithelial cells was injected into ovariectomized SCID female mice bearing an estrogen pellet using a method for heterotransplantation with cultured human endometrial cells [30]. After establishing human ectopic lesions (2 weeks after endometrial cell injection), SCID female mice with human ectopic lesions were treated with oleuropein (25 mg/kg, daily, 21 days) or the vehicle as a control. The luciferase activity image analysis revealed that oleuropein treatment led to significantly less luciferase activity of human ectopic lesions (6-fold, p = 0.012) than the vehicle treatment (Fig. 3d). Therefore, oleuropein also effectively suppressed the growth of human ectopic lesions in SCID mice.

### Oleuropein treatment suppressed proliferation and activated apoptosis in ectopic lesions

**Fig. 4:**
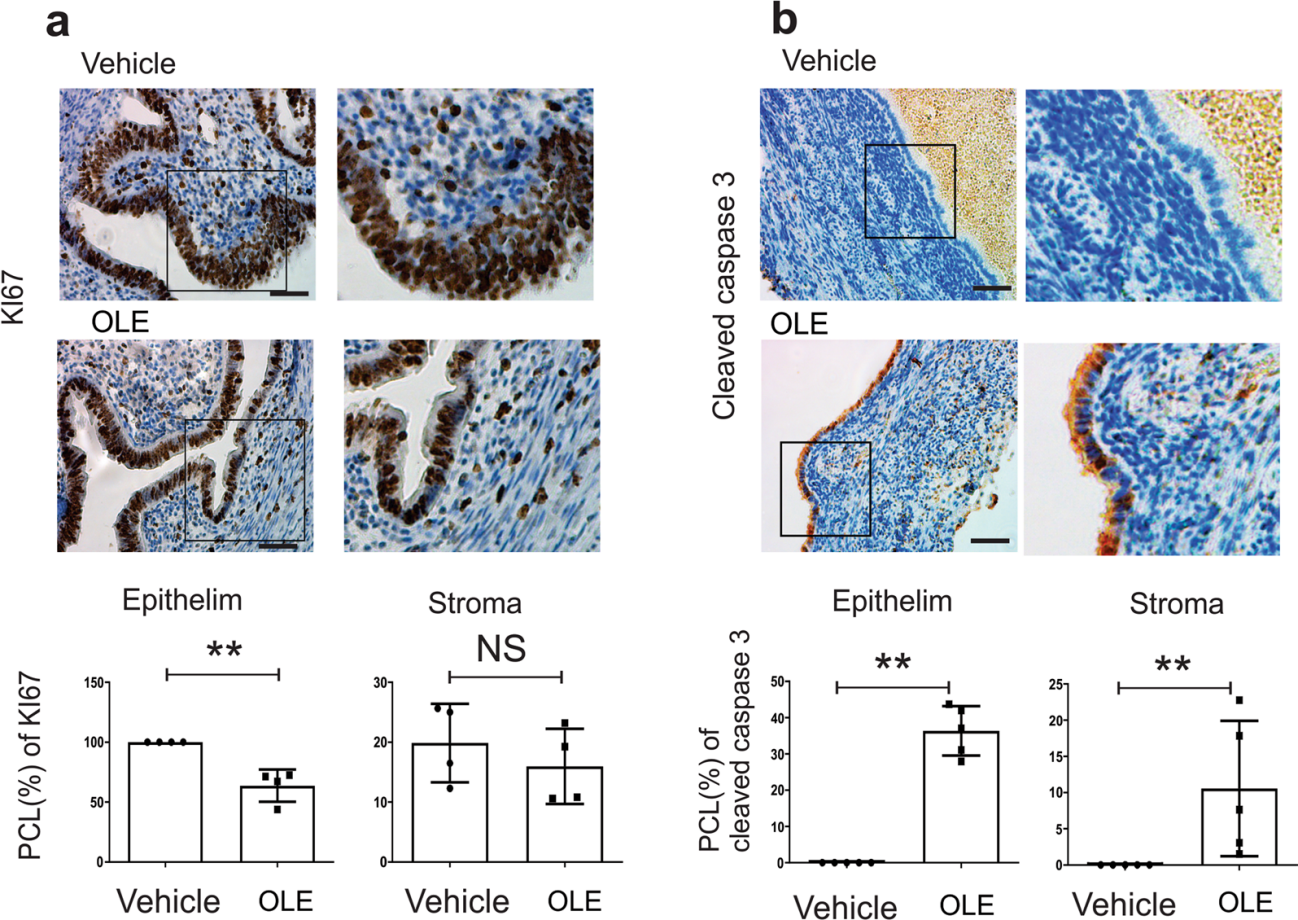
Proliferation and apoptosis signaling in ectopic lesions with oleuropein treatment. **a** Inhibition of proliferation in ectopic lesions by oleuropein. Oleuropein reduced the expression of KI-67 in epithelial, but not stromal, cells in mouse ectopic lesions. **b** Activation of apoptosis in ectopic lesions by oleuropein. Oleuropein increased the expression of the cleaved form of caspase 3 in both epithelial and stromal cells in mouse ectopic lesions. **, P < 0.01; *NS* nonspecific. Scale bar is 50 μm

ERβ increases proliferation and prevents apoptosis in endometriotic lesions to enhance the progression of endometriosis [30]. Since oleuropein specifically inhibits ERβ activity, we determined whether oleuropein suppressed ERβ-mediated proliferation and anti-apoptosis in endometriotic lesions. Immunohistochemistry with Ki-67 revealed that oleuropein treatment led to significantly lower levels of KI-67 in epithelial but not stromal cells of ectopic lesions than the vehicle treatment (Fig. 4a). Therefore, oleuropein effectively inhibited the proliferation of ectopic lesions by suppressing endometriosis progression. Immunohistochemistry with cleaved caspase 3 antibody revealed that oleuropein treatment led to higher levels of the cleaved form of caspase 3 in both epithelial and stromal cells of ectopic lesions than the vehicle treatment (Fig. 4b). Thus, oleuropein reactivated apoptosis in ectopic lesions by inhibiting ERβ and suppressing endometriosis progression.

### Oleuropein did not induce liver cytotoxicity or impact fecundity in female mice

**Fig. 5:**
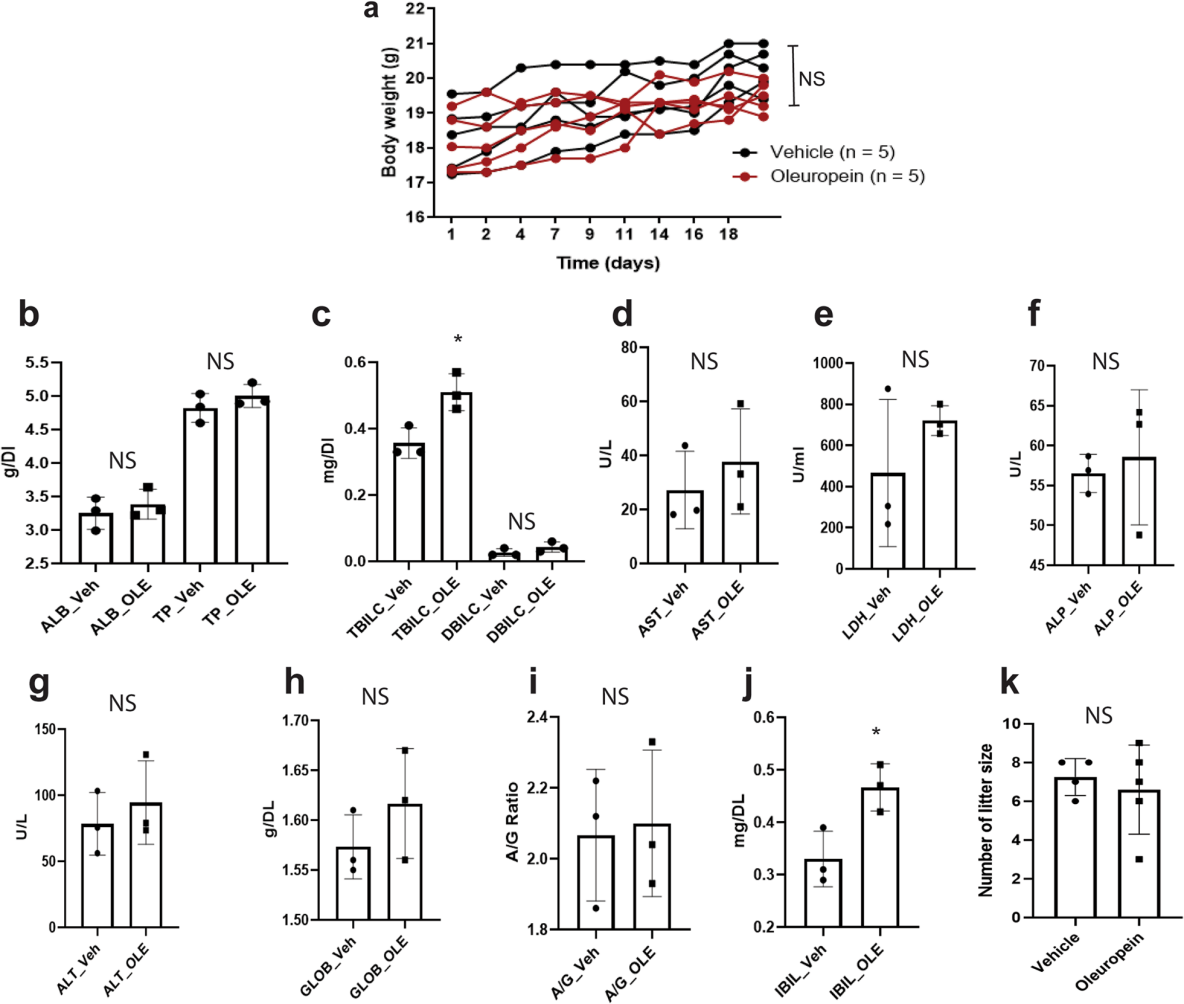
Nontoxicity of oleuropein in mice. **a** No growth retardation of mice was evident with oleuropein (25 mg/kg, once a day) treatment. The body weight of female mice was determined during vehicle versus oleuropein treatment. **b–j**, Liver panel assay of the blood of female mice treated with oleuropein (25 mg/kg, once a day for 21 days) versus the vehicle. Levels of albumin (ALB, **b**), total protein (TP, **b**), total bilirubin (TBIL, **c**), direct bilirubin (DBIL, **c**), aspartate aminotransferase (AST, **d**), lactate dehydrogenase (LDH, **e**), alkaline phosphatase (ALP, **f**), alanine aminotransferase (ATL, **g**), globulin (GLOB, **h**), albumin-globulin ratio (A/G, **i**), and indirect bilirubin (IBIL, **j**) in the blood of C57BL/6J female mice treated with the vehicle (Veh) or oleuropein (OLE). **k** Litter size of C57BL/6J female mice treated with the vehicle or oleuropein (25 mg/kg). *, p < 0.05; *NS* nonspecific

To determine whether oleuropein caused toxicity in mice during the endometriosis treatment, C57BL/6J female mice (8 weeks old) were orally treated with 25 mg/kg oleuropein or corn oil (vehicle control) once a day for 21 days. The oleuropein treatment did not affect body weight (Fig. 5a). We also determined the liver toxicity of oleuropein using a liver panel assay with blood from mice treated with oleuropein versus the vehicle. Oleuropein treatment led to slightly higher levels of TBILC (1.4-fold, p = 0.02) and IBIL (1.3-fold, p = 0.03) in blood than the vehicle treatment (Fig. 5c, j). However, the levels of other enzymes and metabolites in the liver were not higher with oleuropein treatment than with vehicle treatment (Fig. 5b, d–i). Therefore, 25 mg/kg oleuropein does not induce liver toxicity in mice.

ERβ has a role in ovarian function, and ERβ KO mice are partly infertile [40]. Therefore, oleuropein-mediated ERβ targeting therapy might be associated with a potential risk for adverse effects on the fertility of women with endometriosis. To examine whether oleuropein treatment (25 mg/kg) impacts fertility, we examined the fertility of female mice treated with oleuropein versus the vehicle. Oleuropein treatment did not lead to a significantly smaller litter size than the vehicle treatment (Fig. 5k). Furthermore, oleuropein (25 mg/kg) did not cause observable reproductive toxicity in female mice. Oleuropein could be employed for endometriosis treatment without causing liver or reproductive toxicity.

### Oleuropein improved the pregnancy rate of female mice with endometriosis by improving decidualization

**Fig. 6:**
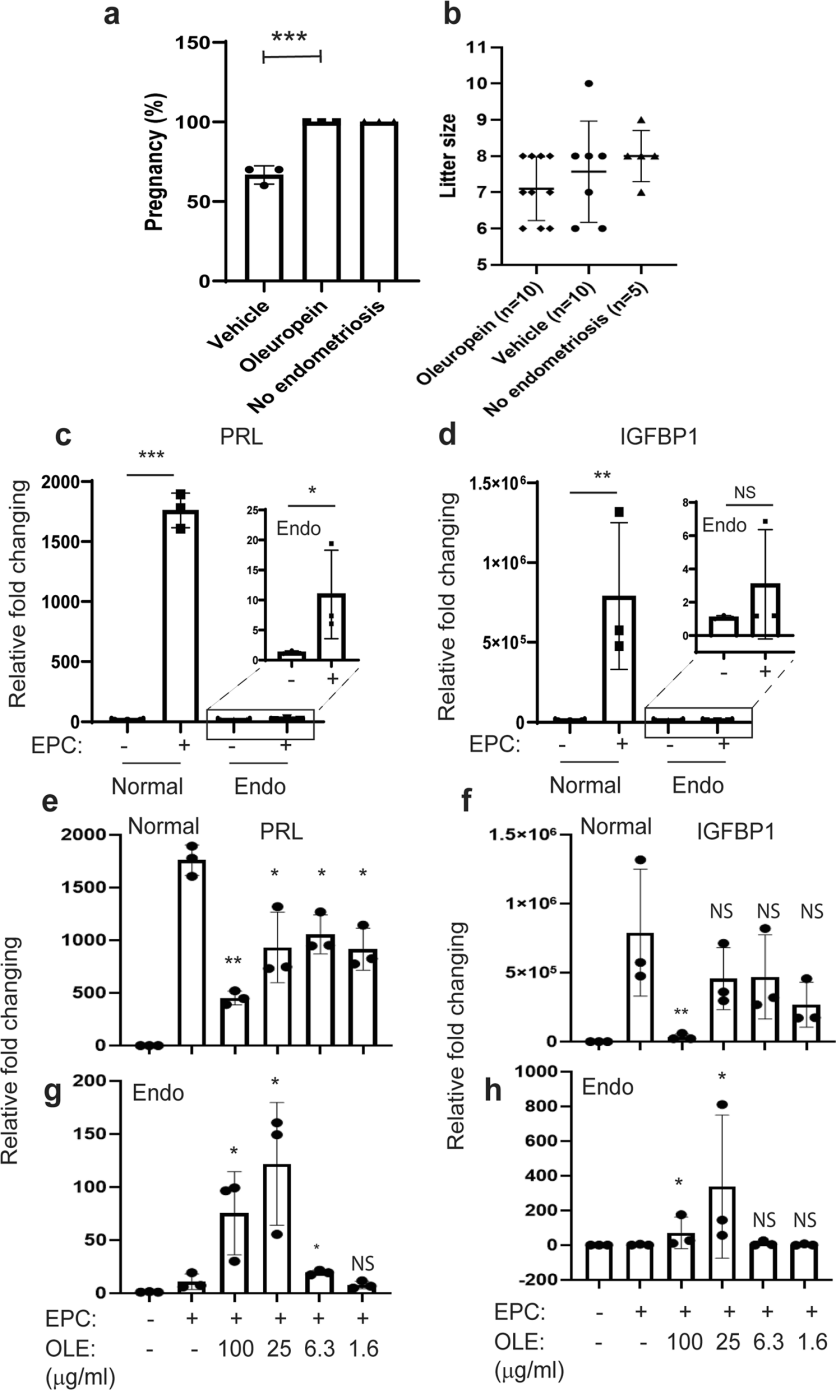
Effect of oleuropein on the pregnancy rate of mice with endometriosis and decidualization of human endometriotic stromal cells. **a** The pregnancy rate of mice with endometriosis treated with vehicle or oleuropein (25 mg/kg). Wild-type mice without endometriosis were employed as the endometriosis control (No endometriosis). **b** The litter size of mice with endometriosis treated with vehicle or oleuropein and the endometriosis control. **c**, **d** The expression levels of PRL (**c**) and IGFBP1 (**d**) in normal human endometrial stromal cells (Normal) and endometriotic stromal cells from ectopic lesions of endometriosis patients (Endo) on the 3^rd^ day after decidualization cocktail (EPC) treatment. **e**, **f** The expression levels of PRL (**e**) and IGFBP1 (**f**) in normal human endometrial stromal cells (Normal) on the 3^rd^ day after decidualization cocktail treatment in the presence of different doses of oleuropein. **g**, **h** The expression levels of PRL (**g**) and IGFBP1 (**h**) in human endometriotic stromal cells (Endo) on the 3rd day after decidualization cocktail treatment in the presence of different doses of oleuropein. *, p < 0.05; **, P < 0.01; ***, p < 0.001; *NS* nonspecific

Current theories for endometriosis-associated infertility are anatomical distortion, endometrial dysfunction, ovulatory dysfunction, and niche inflammation-associated peritoneal or implantation defects [44]. In addition, endometriosis reduced the pregnancy rate in female mice [8]. Our study also reveals that endometriosis led to a lower pregnancy rate (70%) in female mice than in mice without endometriosis (Fig. 6a). In addition, the fertility assay revealed that oleuropein treatment improved the pregnancy rate (100%) of mice with endometriosis (Fig. 6a). However, endometriosis did not affect the litter size of the mice, and oleuropein treatment did not affect the litter size of mice with endometriosis (Fig. 6b).

Endometriosis-associated infertility is partly involved in endometrial dysfunction, such as decidualization defects, and endometrium-specific ERβ-overexpressing mice are infertile due to decidualization defects [30, 39]. Therefore, we determined whether oleuropein treatment can overcome the decidualization defects of human endometriotic stromal cells. Levels of IGF-binding protein-1 (IGFBP-1) and prolactin (PRL), decidual cell markers [28], were much higher in normal human endometrial stromal cells upon treatment with decidualization hormonal cocktail (EPC) than in cells treated with the vehicle (Fig. 6c, d). However, decidualization hormonal cocktail treatment did not induce increased levels of PRL and IGFBP1 in human endometriotic stromal cells compared to normal human endometrial stromal cells (Fig. 6c, d). Therefore, human endometriotic stromal cells have a defect in decidualization progression.

Next, we determined whether oleuropein treatment can rescue the decidualization defect of human endometriotic stromal cells. Treatment with 100 µg/ml oleuropein led to lower levels of PRL (3.9-fold) and IGFBP1 (22.7-fold) in normal human endometrial stromal cells upon decidualization cocktail treatment than those with the vehicle (Fig. 6e, f). However, 25 μg/ml oleuropein slightly reduced the expression levels of PRL (1.6-fold) but did not significantly reduce IGFBP1 expression in normal human endometrial stromal cells (Fig. 6e, f). In contrast with normal human endometrial stromal cells, 100 μg/ml oleuropein led to higher PRL (6.9-fold) and IGFBP1 (23-fold) levels in human endometriotic stromal cells upon decidualization cocktail treatment than those with the vehicle (Fig. 6g, h). Oleuropein (25 µg/ml) showed a marginal effect on the decidualization of normal human endometrial stromal cells. However, 25 µg/ml oleuropein treatment led to significantly higher expression levels of PRL (12.0-fold) and IGFBP1 (310-fold) in human endometriotic stromal cells upon decidualization cocktail treatment than those with the vehicle (Fig. 6g, h). Therefore, oleuropein can partly rescue the decidualization defect and improve the pregnancy rate of female mice with endometriosis.

### Oleuropein reduced dysregulated cytokine levels in endometriotic lesions

**Fig. 7:**
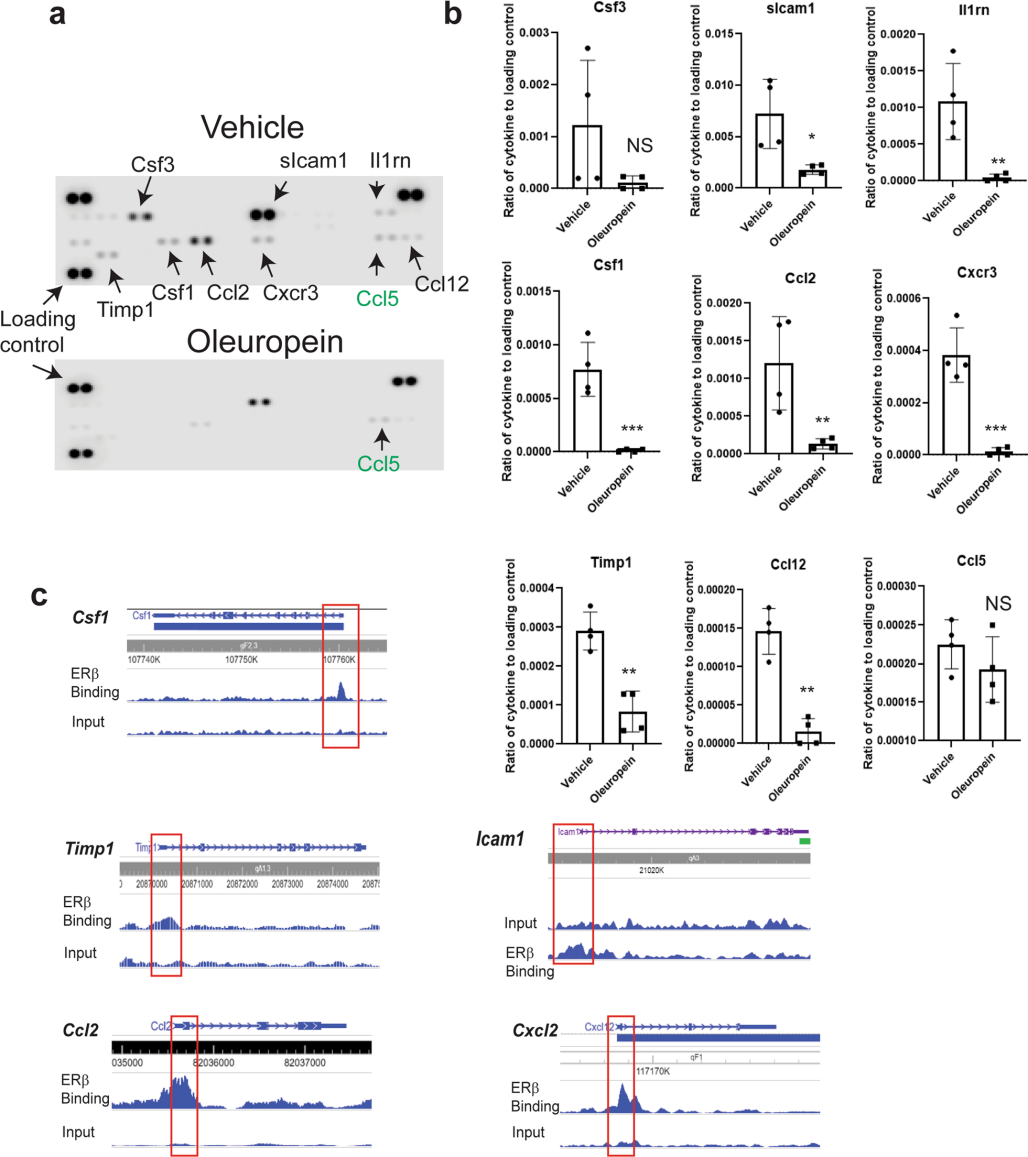
Cytokine profile in ectopic lesions treated with oleuropein or the vehicle.** a** Cytokine levels in ectopic lesions treated with the vehicle and oleuropein (25 mg/kg). **b** Quantification of the cytokine levels in Panel **a**. **c** ERβ binding site on cytokine genes in ectopic lesions of mice with endometriosis. *, p < 0.05; **, P < 0.01; ***, p < 0.001; *NS* nonspecific

Our published study revealed that ERβ is critical in merging estrogen and inflammatory signaling for endometriosis progression because ERβ directly enhances endometriosis-associated cytokines in ectopic lesions [30, 31]. Therefore, we determined whether oleuropein impacts the cytokine profile in ectopic lesions to suppress endometriosis progression. The expression of several cytokines was detected in ectopic lesions treated with vehicle (Fig. 7a). The results show that oleuropein treatment led to significantly lower levels of most cytokines (Csf3, Sicam1, Il1rn, Csf1, Ccl2, Cxcr3, Timp-1, and Ccl12), but not Ccl5, in ectopic lesions than with the vehicle (Fig. 7a, b). Previous studies revealed that the cytokines downregulated by oleuropein are essential in endometriosis progression [3, 37]. Additionally, our endometriotic lesion-specific ERβ-Chromatin ImmunoPrecipitation (ChIP) sequence analysis revealed that Csf1, Timp-1, Icam1, Ccl2, and Cxcl2 are ERβ target genes in ectopic lesions (Fig. 7c) [31]. Endometriotic lesions secrete chemokines into the peritoneal cavity, further stimulating the inflammatory response and release of cytokines to enhance endometriosis progression [43]. Therefore, oleuropein suppresses ERβ target cytokine expression in ectopic lesions to change the endometriosis immune microenvironment, suppressing endometriosis progression and relieving endometriosis-associated infertility.

## Discussion

**Fig. 8:**
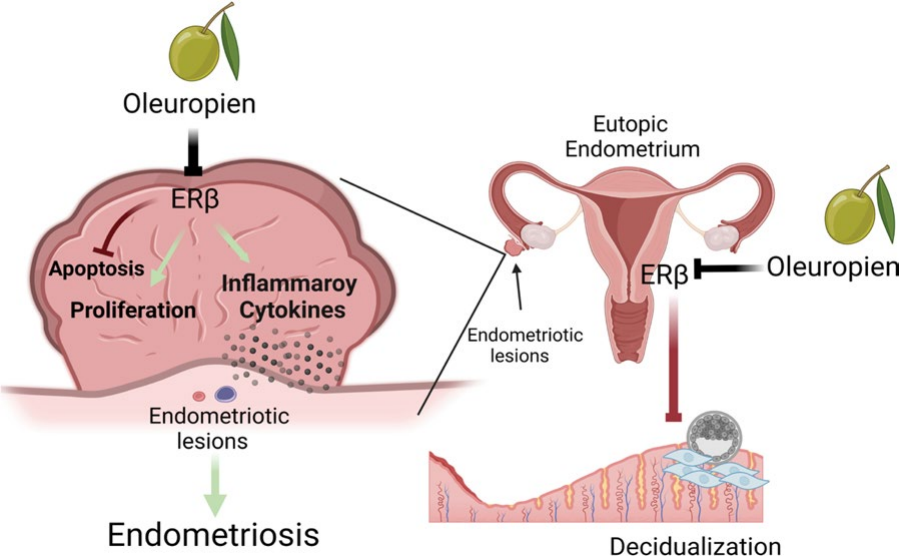
Mechanisms of the oleuropein-mediated suppression of endometriosis. ERβ enhances proliferation, prevents apoptosis, and increases cytokine signaling in ectopic lesions to improve endometriosis [30]. However, oleuropein treatment effectively suppresses ERβ-induced endometriosis-driving cellular pathways in ectopic lesions to suppress endometriosis progression. Furthermore, ERβ overexpression impairs the decidualization processes of the endometrium of mice with endometriosis [30]. Therefore, oleuropein treatment suppresses ERβ function in the endometrium to improve the fertility of mice with endometriosis by rescuing the defect of decidualization of the endometrium

We provide evidence that oleuropein is a novel natural product that selectively inhibits ERβ activity without impacting ERα activity. In this context, oleuropein effectively suppressed the growth of mouse and human ectopic lesions in mice with endometriosis without reproductive toxicity (Fig. 8). Additionally, oleuropein improved the pregnancy rate of mice with endometriosis because it repaired the decidualization defect of the endometrium and reduced the hyperinflammatory state in mice with endometriosis (Fig. 8).

Synthetic selective estrogen receptor modulators (SERMs) have been employed to treat various estrogen-related diseases, including endometriosis [4, 50]. However, the efficacy of SERMs is insufficient to prevent estrogen-related disease progression. Moreover, the clinical usage of SERM medications can have several side effects, such as abnormal vaginal bleeding and pelvic pain [4]. However, the median lethal dose (LD50) value of oleuropein in rats is estimated to be more than 1000 mg/kg. [54]. Olive leaf extract has protective effects against the reproductive toxicity of lead acetate in rats [2]. Thus, oleuropein might be a safer substance than SERMs to treat endometriosis patients without causing reproductive toxicity. Bioavailability studies in humans show that the absorption of olive oil phenols (oleuropein, tyrosol, and hydroxytyrosol) is probably greater than 55–66 mol% and that at least 5% is excreted in urine [64]. The maximal peak of oleuropein and its metabolites in serum and urine is detected in less than 2 h [19]. Therefore, oleuropein can safely and rapidly suppress endometriosis progression.

Our prior study showed that PHTPP, an ERβ-selective antagonist, effectively suppressed endometriosis progression, but PHTPP also partly suppressed uterine ERα activity [30]. Consequently, chronic PHTPP treatment could cause adverse effects in estrogen-targeted tissues. In this context, oleuropein treatment has an advantage for endometriosis treatment over PHTPP because a previous study also revealed that oleuropein is not involved in ERα-mediated regulation of gene expression[58], and the median lethal dose (LD50) value of oleuropein in rats is estimated to be more than 1000 mg/kg [54]. Oleuropein is a nontoxic natural product with no adverse effects generated by inhibiting ERα activity. The metabolism of PHTPP is not clearly described. However, oleuropein is metabolized in vivo into elenolic acid and hydroxytyrosol [53]. Hydroxytyrosol is also one of the major phenolic components in olive leaf extracts and has antiproliferative, antioxidant, and anti-inflammatory effects on various human cancers [18, 60, 63]. The combination of hydroxytyrosol and oleuropein effectively suppressed the migration and invasion of ER-positive breast cancer cell lines compared to their monotherapy [46]. Compared to PHTPP, oleuropein has better suppressive activity of endometriotic tissue progression via the combination of oleuropein and its metabolites, such as hydroxytyrosol.

Letrozole, an aromatase inhibitor, has been employed to effectively treat endometriosis and relieve endometriosis-associated pain in combination with gestagens, oral contraceptives, or gonadotropin-releasing hormone (GnRH) agonists [25, 47]. Additionally, the combination of a GnRH agonist and letrozole has been used to treat infertility caused by endometriosis [59]. However, long-term use of aromatase inhibitors increases the risk of osteoporosis and bone fractures [55]. Unlike letrozole, oleuropein has critical effects on the formation and maintenance of bone and can be used as an effective remedy to treat osteoporosis symptoms [29]. Therefore, oleuropein may have a better beneficial effect in endometriosis patients than letrozole.

Olive leaf extract has protective effects against the reproductive toxicity of lead acetate in rats [2]. In addition, olive leaf extracts have various beneficial effects on human health, such as antimicrobial, antiviral, antioxidant, anti-inflammatory, antiaging-associated neurodegeneration, and anticancer effects [9, 10, 17, 65]. Furthermore, oleuropein is a major component of olive leaves [up 19% (w/w)] [42]. Therefore, olive leaf oleuropein and oleuropein-rich food could be employed as nutraceutical therapies to treat endometriosis progression for improved efficacy and reduced adverse effects compared with the current hormonal treatments for endometriosis.

Oleuropein suppressed ERβ activity induced by estradiol through the inhibition of nuclear translocation of ERβ. In addition to preventing the nuclear localization of ERβ, oleuropein suppressed the TNFα-induced phosphorylation of Akt and p44/p42 MAP kinase and attenuated TNF-α-stimulated M-CSF and IL-6 release [33]. In addition, Akt enhances ERβ activity in breast cancers [21]. The functional connection between oleuropein, kinase signaling, and ERβ will be further investigated to define the molecular mechanism of oleuropein-mediated suppression of endometriosis.

Oleuropein is metabolized in vivo into oleanolic acid and hydroxytyrosol by β-glucosidase and esterase activity in humans and mice [53]. Hydroxytyrosol is also one of the major phenolic components in olive leaf extracts and has antiproliferative, antioxidant, and anti-inflammatory effects on various human cancers [18, 60, 63]. Furthermore, the combination of hydroxytyrosol and oleuropein led to effectively less migration and invasion of ER-positive breast cancer cell lines than monotherapy [46]. Therefore, in this context, the efficacy of the combination of oleuropein and hydroxytyrosol could be investigated to define whether the combination has a better beneficial effect than oleuropein monotherapy.

The main strength of this study is that it provides a novel strategy to target ERβ therapeutically with oleuropein as a nonhormonal therapy for endometriosis. The weaknesses of this study are that oleuropein is not an unknown material, and the molecular mechanism of oleuropein-mediated ERβ inhibition was not defined completely.

## Conclusions

The present study identified that oleuropein selectively inhibited ERβ-mediated endometriosis driving cellular pathways (such as proliferation, anti-apoptosis, and inflammation) to suppress endometriosis progression without reproductive toxicity. Additionally, oleuropein improves the fertility of female mice with endometriosis by partly recuring the impaired decidualization. Therefore, oleuropein is a new nutraceutical product for use in nonhormonal therapy for endometriosis.

## Supplementary Information

**Additional file 1. Fig. S1.** a-d Expression profile of phospho kinases involved in AKT (a), JAK/STAT (b), NF-κB (c), and TGFβ (d) pathways in ectopic HESCs treated with vehicle or 10 and 100 nM OLE for 24 h.

## Data Availability

The datasets used and/or analyzed during the current study are available from the corresponding author on reasonable request.

## Abbreviations

ER.: Estrogen receptor
A/G: Albumin-globulin ratio
ALP: Alkaline phosphatase
AST: Aspartate aminotransferase
ATL: Alanine aminotransferase
Ccl12: C-C motif chemokine ligand 12
Ccl2: C-C motif chemokine ligand 2
Ccl5: C-C motif chemokine ligand 5
ChIP: Chromatin immunoprecipitation
COX-2: Cyclooxygenase-2
Csf1: Colony stimulating factor 1
Csf3: Colony stimulating factor 3
Cxcr3: C-X-C motif chemokine receptor 3
DBIL: Direct bilirubin
DMEM: Dulbecco's minimum essential medium
E2: Estradiol-17β
GnRH: Gonadotropin-releasing hormone
HSD17B2: 17β-Hydroxysteroid dehydrogenase-2
IBIL: Indirect bilirubin
IGFBP-1: Insulin-like growth factor binding protein-1
IHEECs: Immortalized human endometrial epithelial cells
IHEECs:ERΒ: Immortalized human endometrial epithelial cells overexpressing ERβ
IHESCs: Immortalized human endometrial stromal cells
Il1rn: Interleukin 1 receptor antagonist
IL-6: Interleukin 6
IVIS: In vivo image system
M-CSF: Macrophage colony-stimulating factor
OLE: Oleuropein
PGE2: Prostaglandin E2
PRL: Prolactin
SCID: Severe combined immunodeficiency
Sicam1: Soluble intercellular adhesion molecule-1
TBIL: Total bilirubin
Timp-1: TIMP Metallopeptidase Inhibitor 1
TP: Total protein
