# Supplementary figures and images for: Oleuropein suppresses endometriosis progression and improves the fertility of mice with endometriosis
Source: J Biomed Sci. 2022 Nov 22;29:100. doi: 10.1186/s12929-022-00883-2 (PMC9682776; doi:10.1186/s12929-022-00883-2)

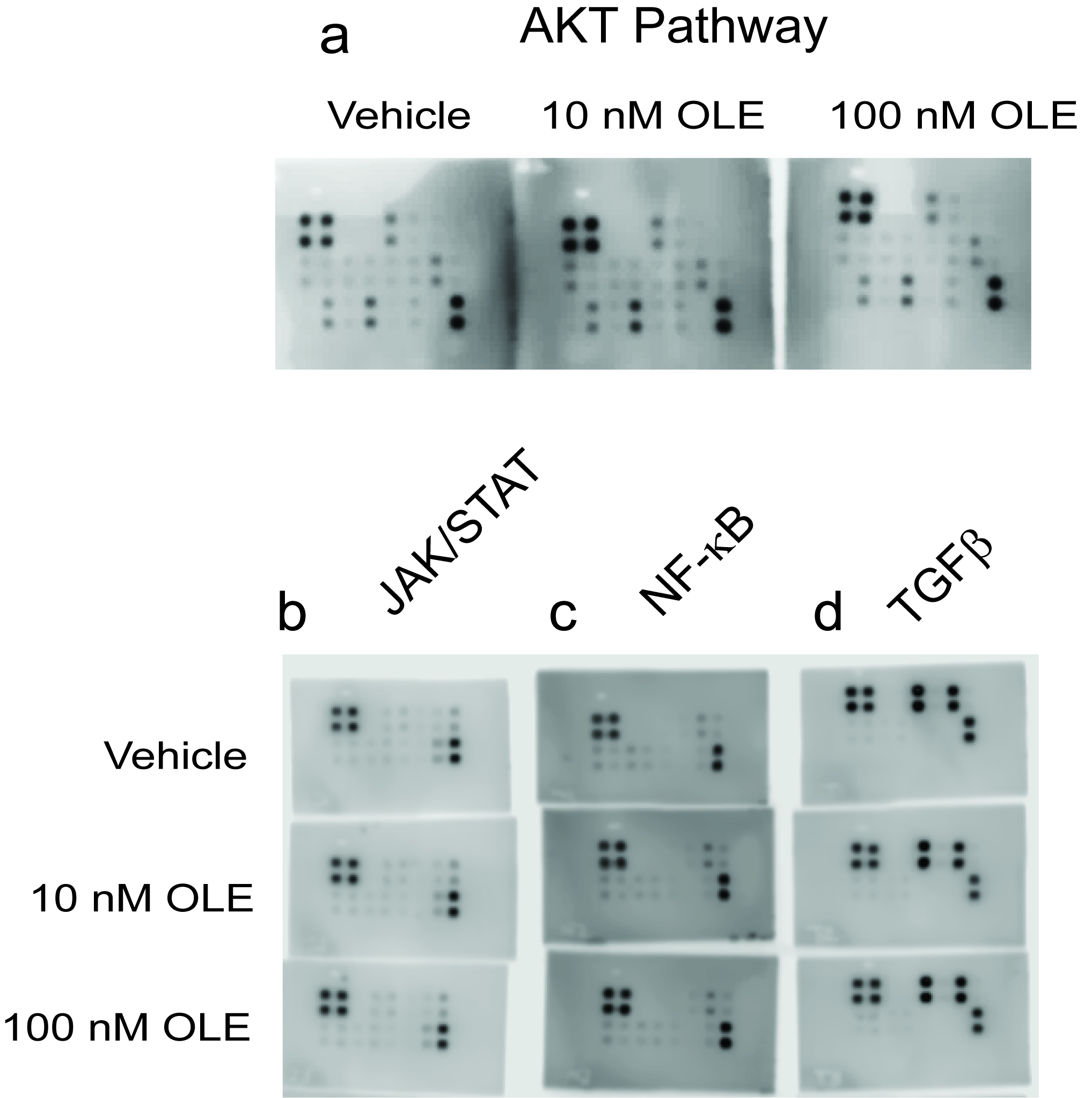

Supplement: Supplementary file 1 — Additional file 1. Fig. S1. a-d Expression profile of phospho kinases involved in AKT (a), JAK/STAT (b), NF-κB (c), and TGFβ (d) pathways in ectopic HESCs treated with vehicle or 10 and 100 nM OLE for 24 h. [file 12929_2022_883_MOESM1_ESM.tif]
